# The Effect of Microfluidization Pressure and Tocopherol Content on the Retention of Vitamin A in Oil-In-Water Emulsions

**DOI:** 10.3390/foods10030504

**Published:** 2021-02-26

**Authors:** Shahin Banasaz, Ksenia Morozova, Giovanna Ferrentino, Matteo Scampicchio

**Affiliations:** Faculty of Science and Technology, Free University of Bozen-Bolzano, Piazza Università 5, 39100 Bozen-Bolzano, Italy; Shahin.banasaz@natec.unibz.it (S.B.); Giovanna.ferrentino@unibz.it (G.F.); Matteo.scampicchio@unibz.it (M.S.)

**Keywords:** encapsulation, emulsion, vitamin A, shelf-life study, stability

## Abstract

This work investigates the oxidative stability of vitamin A encapsulated in oil-in-water emulsions, which were prepared by using a microfluidizer. All emulsions were prepared with a fixed content of vitamin A (525 µM), corn oil (10%), water (90%), and whey protein (2%), but varying two main factors: the microfluidizer pressure (10, 50, 100, 200 MPa) and the amount of α-tocopherol (0, 0.25, 0.50, 1.00 mg/g). The content of vitamin A before and after the microfluidization process, and during the subsequent five weeks of storage at 40 °C were determined by HPLC-DAD. The results of the analysis of variance performed either on the data obtained before and after the microfluidization process or during the storage showed that the highest stability of vitamin A was obtained with the highest content of α-tocopherol and with an applied pressure between 100 and 200 MPa. The highest stability was explained by the smaller particle size of the resulting oil droplets. However, high pressures (200 MPa) showed a negative effect on vitamin A retention. These results could be useful for future formulations of retinoids.

## 1. Introduction

The encapsulation of lipophilic vitamins in water-based beverages is a challenge for many foods and supplement manufacturers. A relevant example is offered by retinoids, a class of chemical compounds that are vitamers of vitamin A. Retinol and its esters (i.e., retinyl acetate or palmitate) have high insolubility in water or glycerol. As vitamin A deficiency nowadays affects about one-third of children under the age of five around the world, it is important to develop novel solutions for improving its stability in water-based beverage systems [1,2,3,4]. To prevent the problem of vitamin A degradation, emulsification is a common strategy.

In emulsions, one phase is dispersed in another with an addition of surfactants. The two most common types of emulsions are oil-in-water (O/W) (e.g., milk and mayonnaise) and water-in-oil (W/O) (e.g., butter and margarine). An emulsion can be an ideal encapsulation and delivery system for the incorporation of bioactive compounds including carotenoids, essential oils, and fatty acids into the lipid or aqueous phase. As a result, their bioavailability can be increased. Because of their smaller size, nanoemulsions can be more stable against coalescence, flocculation, and gravitational separation [5,6].

The encapsulation of vitamin A in nanoemulsions is challenging because of low water solubility and sensitivity to oxidation [7]. For instance, the limitations of using vitamin A in microemulsions were investigated by Hwang et al. [8], who showed that only 59% of the initial retinoic acid was retained less than 7 h after emulsion preparation. In another study by Tanglao et al. [9] vitamin A in form of retinyl acetate was encapsulated in virgin coconut oil-in-water emulsion stabilized by whey protein as an emulsifier. In this study, the emulsions were prepared using different homogenization speeds: 720, 846.7, and 955.8 rpm. The results obtained by different scanning calorimetry and microscopy analysis showed thermal stability of vitamin A increased by encapsulation by the nanoemulsion technique [9].

Many emulsion characteristics i.e., stability, rheology, appearance, color, texture, and shelf-life, depend on droplet size and distribution. Nanoemulsions are thermodynamically unstable systems with a mean diameter ranging between 20 to 100 nm and even up to 500 nm with a milky appearance. [10,11]. Nanoemulsion preparation can be performed by two different approaches, high-energy and low-energy methods, respectively. In the high-energy method, nanoemulsions can be formed by the application of sonication or microfluidization.

Currently, one of the most advanced solutions to improve the stability of vitamin A is the use of emulsions prepared by microfluidization [12,13,14]. In this method, high shear stress can form small droplets from a previously prepared coarse emulsion [15]. Typically, bioactives like vitamin A are dispersed in the oil phase. This is coarsely mixed with water and an emulsifier. Finally, the slurry is passed through the small inlet orifice at the microfluidizer. The breaking of liquids with high-intensity energy systems may lead to nanoemulsions. Nanoemulsions have the advantage to be composed of extremely fine oil droplets, with an average diameter lower than 500 nm, at low costs and large-scale production [16,17,18,19,20,21,22]. The preparation of nanoemulsion delivery systems for the encapsulation of lipophilic bioactives has been previously reported for vitamin D, E, and β-carotene [23,24,25,26,27,28,29,30,31]. However, the small size droplets in the transparent nanoemulsions system may lead to the chemical degradation of the encapsulated components due to exposure to the UV and visible light. This light sensitivity can easily promote chemical degradation reactions. One of the further steps to improve the chemical stability of the encapsulated sensitive components, such as vitamin A, within nanoemulsions is the addition of antioxidants [32]. Lipophilic antioxidants, such as tocopherols, have shown better results in polar systems such as O/W emulsions in comparison to water-soluble antioxidants because of their location in the oil phase where oxidation propagates [33].

Nevertheless, the reduction of the oil droplet size down to the nanoscale poses an interesting question. From one side, the higher interfacial area in nanoemulsion systems would increase the amount of emulsifiers in contact with the lipid phase and increase the efficiency of protein in stabilizing the emulsion against both creaming and oil oxidation [16]. Moreover, the higher concentration of emulsifiers and antioxidants surrounding the droplets would, in turn, explain the resulting higher stability of vitamin A. At the same time, however, an excessive reduction of the oil droplet size could decrease the vitamin A stability simply because the higher surface areas of the O/W interphase will also enhance the oxygen diffusion and, thus, the occurrence of oxidation reactions [34,35]. Which of the two cases prevails, cannot be easily predicted. One of the reasons is that during the preparation of nanoemulsions by microfluidizer, the high temperatures and applied high shears and impact forces may further degrade the emulsifiers and/or the antioxidants, vanishing their protective effect. Because of the system complexity, the capacity to predict the long-term chemical stability of vitamin A, encapsulated in O/W nanoemulsion systems is still limited. Due to such complex behavior, it is of great importance for the food industry to develop new rapid methods that could predict in short times the expected stability of an emulsion.

For this reason, this work aims to investigate if the retention of vitamin A in emulsions systems during long storage times could be predicted by a simple microfluidizer test. In other words, this study aims to correlate the vitamin A loss observed during long storage studies of the vitamin A loss observed during simple trials performed before and after microfluidization. Such correlation could be of great importance for the food industry because it would suggest the possibility to predict the long storage stability of vitamin A based on quick trials performed immediately before and after microfluidization.

Although several studies examined the effect of microfluidizer pressure on both the physical and chemical stability of nanoemulsions, there are no studies on the effect of changing microfluidizer pressure up to 200 MPa (which generates different particle size distribution) on the retention of vitamin A encapsulated by whey protein isolate (WPI) and second on the varying antioxidant concentration on the vitamin A retention during a storage test. Overall, this work will provide new insights into the formulation of stable vitamin A nanoemulsion systems. Furthermore, this study will also investigate the possibility to predict the long-term stability of emulsions by simple trials obtained just before and after microfluidization.

## 2. Materials and Methods

### 2.1. Chemicals

Whey protein isolate was purchased from Fonterra Coöperatie U.A. Vitamin A was obtained from the DSM company (Nutritional products holding AG, Bazel, Switzerland). Corn oil and α-tocopherol were purchased from Sigma Aldrich (Milano, Italy). All reagents were of chemical grade. Phosphate buffer (10 mM, pH = 7.0) was used to prepare all solutions and emulsions.

### 2.2. Emulsions Preparation

The oil phase was prepared by adding 0.5% *w/w* of vitamin A in corn oil (525 µM, final concentration), with or without the addition of α-tocopherol (0.0, 2.5, 5.0, and 1.0 mg/g emulsion, final concentration). The mixture was heated at 50 °C for 5 min and then stirred at room temperature for about 1 h to ensure full dissolution. During this step, each sample was flushed with nitrogen to avoid oxidation. The aqueous phase was prepared by dispersing 2% (*w/w*) whey protein isolate (WPI) in an aqueous buffer solution (10 mM phosphate buffer, 0.01% (*w/w*) sodium azide, pH 7.0) to ensure microbiological stability of the studied emulsions.

Oil-in-water (O/W) nanoemulsions were prepared by homogenizing 10% (*w/w*) of the oil phase with 90% (*w/w*) of the aqueous phase at room temperature (25 °C). Preliminarily, a coarse emulsion was prepared using an Ultra-Turrax (Model T25 digital, IKA, Königswinter, Germany) homogenizer operating at 18,000 rpm for 2 min. Then, the coarse emulsion was passed through a microfluidizer (Model 101, Microfluidics, Newton, MA, USA) equipped with an auxiliary processing diamond channel of 200 µm, which act as premixing, followed by a Z-type diamond cell of 87 µm channel diameter (H30Z 200 µm and G10Z 87 µm). For each preparation, pressures of 10, 50, 100, and 200 MPa were applied for three cycles. Each resulting emulsion (about 100 mL) was divided into two aliquots, placed in falcon tubes covered by aluminum foil to prevent photo-oxidation, and, finally, stored at +40 °C in the dark.

### 2.3. Colloidal Stability

The effect of WPI concentration and the number of microfluidizer cycles (1 to 4) on the physical stability of emulsions was tested by multiple light scattering measurements performed with an optical analyzer Turbiscan^®^ Lab Tower (Formulation, L’Union, France). The instrument is equipped with six stations, where 20 mL vials are loaded. The reading head is composed of a pulsed near-infrared light source (λ = 880 nm) and two synchronous transmission and backscattering detectors. The backscattering detector, located at 45° from the incident beam, detected the light scattered backward by the sample. The backscattering spectra of the samples were acquired every 20 min along the entire sample height for 24 h at 25 °C. From the backscattering spectra the Turbiscan stability index (TSI) was calculated using the Equation (1).

(1) $$\mathrm{TSI}\;=\;\sqrt{\frac{\sum_{i=1}^{n}{\left(x_{i}-x_{\mathrm{BS}}\right)}^{2}}{n-1}}$$

where x_i_ is the average backscattering for each minute of measurement, x_BS_ is the average x_i_, and n is the number of scans [27,36].

### 2.4. Droplet Size Distribution

The droplet size distribution (DSD) of the prepared emulsions was determined by the static light scattering technique using a Mastersizer Hydro 3000 (Malvern Instruments Ltd., Malvern, Worcestershire, UK). The measurement was done on fresh emulsion after the preparation and during storage with the following parameters: obscuration ~9%, a refractive index of 1.52, and absorption index of 0.01. The surface mean diameter D [4,3] and the volume mean diameter D [3,2] were reported as mean and standard deviation from a total number of nine measurements. Dv (10) and Dv (90) values were also measured and reported to indicate the width of the size droplet distribution.

### 2.5. Quantitative Analysis of Vitamin A by High-Performance Liquid Chromatography

The determination of vitamin A content was performed by HPLC according to the method of Gatti et al. (1999) with some modifications [37]. Briefly, an emulsion sample was accurately weighed (500 mg) into a falcon tube and extracted with 4 mL of a solvent system composed of acetonitrile-ethanol-acetic acid (70:20:10, *v*:*v*:*v*) and extracted by ultrasounds (35 kHz) for 5 min. The supernatants were separated by centrifugation for 6 min at 3500 rpm and filtered with a 0.45 µm syringe filter. Then, 100 µL of this solution was diluted with 990 µL volume of mobile phase (ethanol, 98%, HPLC grade) and used for later HPLC determination.

The HPLC determination was performed using Agilent 1260 Infinity Binary LC system equipped with a binary high-pressure gradient pump with high-pressure switching valves, online degasser unit, a high-sensitivity ultraviolet detector, high-speed drive autosampler with a 20 μL loop, which accommodates 100 samples at a time with direct access rack system and large capacity column oven. All of the system was controlled by ChemStation software v.A.01.03. The separation was carried out with a Thermo Fischer ODS Hypersil 125 × 4 mm column (pore size 5 µm). The mobile phase consists of isocratic elution using 98% methanol with 2% water. The flow rate of 1 mL/min, injection volume of 20 µL, column temperature 25 °C, and detection wavelength 326 nm were set. Overall run time was 10 min. All solutions were degassed and filtered through a 0.45 μm pore size filter. The instrument was calibrated and qualified before the analysis by using these chromatographic conditions on a standard solution of vitamin, separately. In addition, the system suitability parameters such as tailing factor (T) and theoretical plates (N) were also calculated with respect to the retinyl acetate standard solution (10 µM).

### 2.6. Statistical Analysis

The study was carried out using a full factorial design with two (Section 3.1) and three (Section 3.2) independent variables, respectively, the pressure of the microfluidizer (10, 50, 100, and 200 MPa), the concentration of α-tocopherol (0.00, 0.25, 0.50, and 1.00 mg/g), and time of storage (0, 1, 2, 3, 4, and 5 weeks), with each experiment repeated in triplicate. The dependent variable was the content of vitamin A or its % loss. The statistical analysis was performed by the IBM SPSS statistics software, v.25. The optimization of parameters and desirability plot was done using Design-Expert software v.12.

## 3. Results and Discussion

### 3.1. Physical Properties of the Emulsions

#### 3.1.1. Colloidal Stability

**Figure 1:**
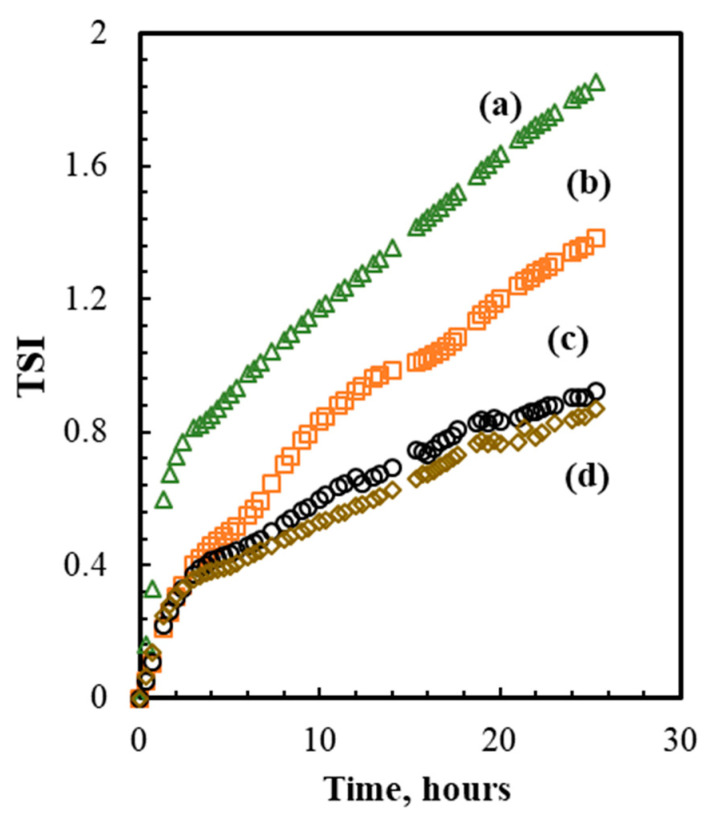
Turbiscan stability index (TSI) obtained along the emulsion samples prepared with 2% (*w/w*) WPI for one (**a**); two (**b**); three (**c**) and four (**d**) microfluidizer cycles at a pressure of 100 MPa.

Figure 1 shows the Turbiscan stability index (TSI) of nanoemulsions containing 2% (*w/w*) of whey protein isolate prepared with one, two, three, and four cycles of microfluidizer process at 100 Mpa. The results showed that the emulsions prepared with one and two cycles were not stable as the TSI index was changing rapidly as observed from the slope of the curves (a) and (b). Instead, after three and four cycles, the emulsions showed good physical stability. The outlet temperature after the first cycle was 33 °C and linearly increased up to 82 °C after three cycles. Based on the results of TSI, the minimum number of cycles needed to produce a stable emulsion and avoid the degradation of vitamin A due to exposure to high temperature was set equal to three. These conditions were chosen for further experiments for the encapsulation of vitamin A.

**Figure 2:**
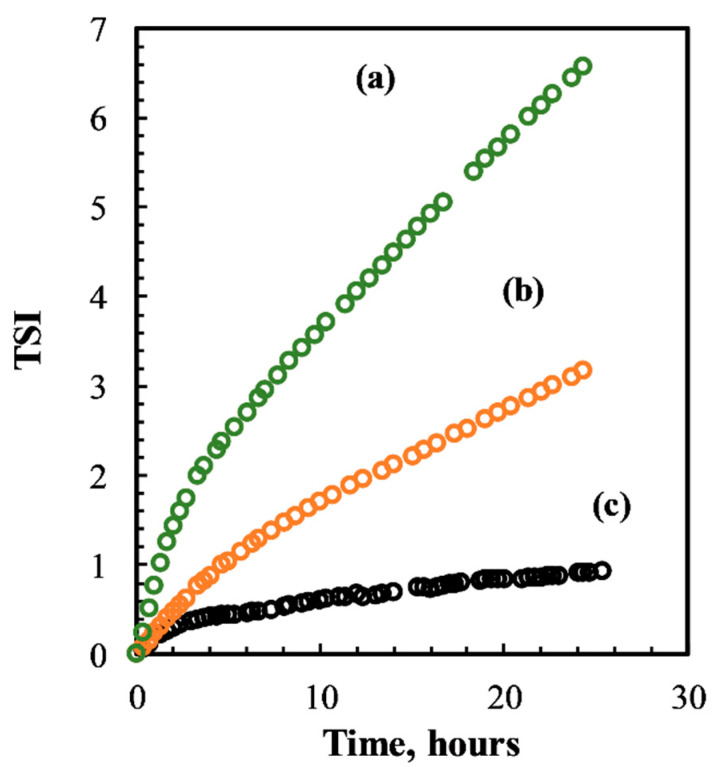
Turbiscan stability index (TSI) obtained for the emulsion samples prepared with 0.1% (**a**); 1% (**b**) and 2% (**c**) WPI (*w/w*) at a pressure of 100 MPa and three microfluidizer cycles.

Besides the number of cycles, preliminary experiments were also carried out to assess the effect of WPI concentration on the physical stability of the emulsions prepared at 100 MPa and three microfluidizer cycles. Figure 2 shows the effect of increasing concentration of WPI on the emulsion physical stability expressed in terms of TSI. It was evident that WPI concentrations lower than 2% (*w/w*) did not induce any physical stability to the emulsions. Thus, 2% (*w/w*) was considered the best option for further experiments.

#### 3.1.2. Droplet Size Distribution

**Figure 3:**
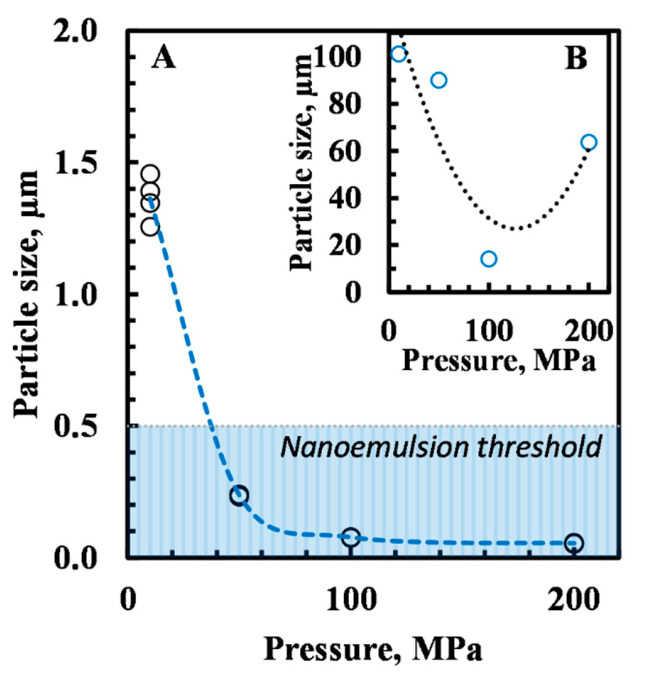
Surface mean diameter D [3,2] of emulsions prepared with 2% (*w/w*) WPI as a function of applied pressure at day 1 (**A**) and after 5 weeks of storage (**B**).

The volume-based droplet size distribution (DSD) of emulsion samples was determined on the same day of emulsion preparation and at the end of storage at 40 °C to monitor the effect of microfluidizer pressure on droplet size. The changes were monitored using surface mean diameter D [3,2], which is sensitive to the presence of small particles (Figure 3). After preparation, the emulsion particle size decreased significantly by increasing the pressure from 10 MPa to 50 MPa (Figure 3A). All emulsions prepared by 50–200 MPa could be considered nanoemulsions. However, during five weeks of accelerated storage test at 40 °C, the particle size of emulsion samples increased. As a result, only samples prepared using a pressure of 100 MPa maintained the physical stability after five weeks (Figure 3B). The other samples remained stable only up to three weeks.

### 3.2. Effect of Microfluidization Process on Vitamin A Loss

Emulsions samples were prepared by following a completely randomized design, by varying two independent factors, namely (1) the applied microfluidizer pressure and (2) the content of α-tocopherol. Table 1 and Table 2 (column named “week 0”) shows the effects of these two factors on the resulting vitamin A loss just after microfluidization. In details, the microfluidizer pressure varied at four levels (10, 50, 100, and 200 MPa) and the α-tocopherol content consisted of four concentrations (0.00, 0.25, 0.50, and 1.00 mg/g).

A two-way analysis of variance revealed that all the main effects were statistically significant at the 0.05 level. The content of α-tocopherol was the most important variable explaining 85% of the total variance. An F ratio of F3,16 = 117 with *p* < 0.001 indicated a significant difference between mean retention of vitamin A in samples with 0.00 mg/g (416 µM, D), 0.25 mg/g (450 µM, C), 0.50 mg/g (480 µM, B), and 1.00 mg/g (503 µM, A) of α-tocopherol. The letters correspond to the ANOVA analysis results at Tuckey HSD *p* < 0.05.

**Figure 4:**
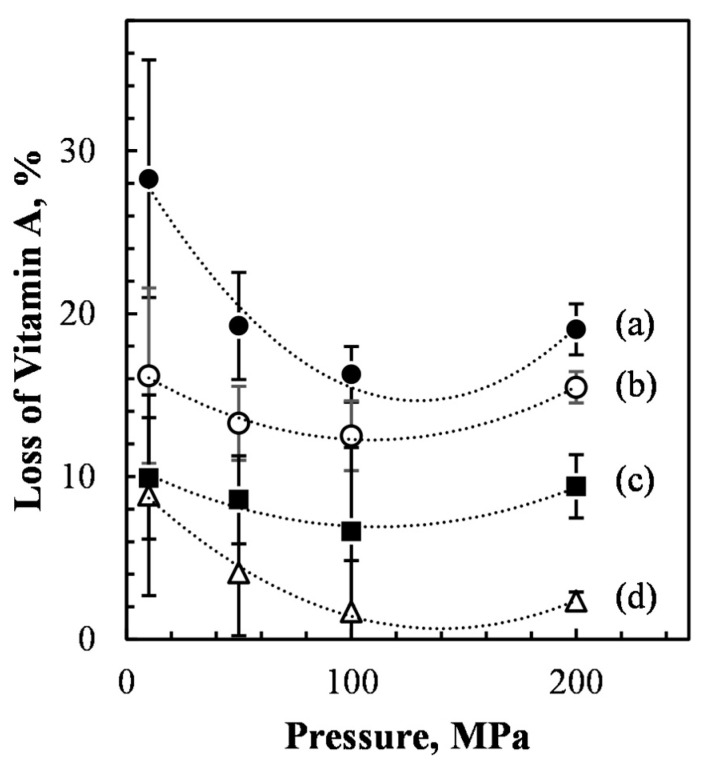
Effect of the applied pressure on the percentage loss of vitamin A, measured just after the microfluidization step compared to the coarse emulsion before microfluidizer process, for samples containing increasing concentrations of α-tocopherol: (**a**); 0 mg/g; (**b**) 0.25 mg/g; (**c**) 0.50 mg/g; (**d**) 1.00 mg/g.

The effect of pressure was also significant (F3,16 = 17, *p* < 0.01), indicating that the influence of the applied microfluidizer pressure of 10 MPa (mean retention 442 µM, B), 50 MPa (466 µM, A), 100 MPa (484 µM, A), and 200 MPa (464 µM, A) was also rather important. Figure 4 shows that the effect of pressures on vitamin A loss follows a quadratic trend, with a local minimum.

Although the pressure of the microfluidizer explained a smaller percentage of the total variance (12%), an increase of pressure from 10 to 100 MPa significantly reduced the loss of vitamin A during the microfluidization process from 28 to 16%. Nevertheless, the applied pressure of 200 MPa had a negative effect on vitamin A stability (vitamin A loss of 19%). This may be due to the exposure to high temperatures during the emulsion preparation. This may have caused the degradation of vitamin A and loss of its activity. Finally, the interaction effect of pressure and α-tocopherol content was not significant, with an F ratio of F (9,16) = 2, *p* > 0.05.

**Figure 5:**
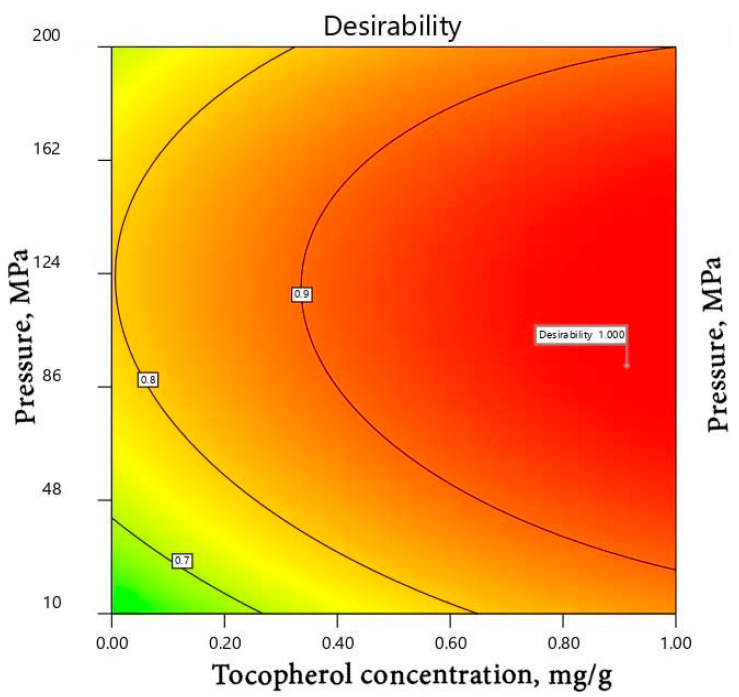
Desirability plot reporting the effects of the applied pressure and the concentration of α-tocopherol on the resulting percentage loss of vitamin A, measured after the microfluidization step of emulsion preparation.

Optimization of the microfluidization parameters is presented in a two-dimensional contour plot in Figure 5. 

The plot shows the effects of microfluidizer pressure and α-tocopherol content on vitamin A loss during emulsion preparation. The contour lines are iso-response values. Accordingly, the best combination of pressure and α-tocopherol concentration is enclosed in the contour line having the lowest % loss value. Thus, optimal process parameters (i.e., minimal vitamin loss) were obtained at pressures between 100 and 200 MPa and α-tocopherol concentrations above 0.7 mg/g. With such conditions, the percentage loss of vitamin A can be neglected (1%).

The results presented here extend the works of Horn et al. [38,39] on the lipid oxidation of fish O/W emulsions. In those works, high pressures improved the adsorption of whey proteins, which formed a protective layer surrounding the O/W interface [38,39]. The possible explanation can be that the concentration of whey protein molecules on the surface of oil droplets may have increased due to the application of higher pressure causing the increase of surface hydrophobicity [40,41]. This can explain the higher retention of the encapsulated vitamin A with the increase of pressure from 10 to 100 MPa. However, when very high energy (microfluidizer pressure) is applied, large emulsifying molecules, such as WPI, cannot quickly be adsorbed at the newly formed interface. This explains the lower retention of the vitamin in samples prepared with 200 MPa [10,11,15].

The effect of higher pressures to reduce the oil droplet size was confirmed by laser diffraction analysis. The results led to particle sizes distribution in the ranges between Dv (10) 0.87 and Dv (90) 14 µm for 10 MPa; Dv (10) 0.059 µm, and Dv (90) 1.29 µm for 200 MPa, with evolution to smaller sizes when the microfluidizer pressure increased. The surface-based diameter D [3,2] of samples decreased by an increase in the microfluidization pressure from 1.36 ± 0.1 µm for 10 Mpa, 0.24 ± 0.06 µm for 50 MPa, 0.08 ± 0.04 µm for 100 MPa and 0.05 ± 0.02 µm for 200 MPa. Additionally, the mean volume-based diameter D [4,3] decreased as the microfluidizer pressure increased, resulting in 11 ± 2 µm for 10 MPa, 0.39 ± 0.02 µm for 50 MPa, 0.28 ± 0.01 µm for 100 Mpa, and, finally, 0.19 ±0.04 µm for 200 MPa. These results confirmed the works of Sørensen et al. where the effect of high-pressures increased the oxidative stability of fish-oil-enriched milk emulsions by reducing the size of the oil droplets [42,43].

However, the effect of high pressure on the oxidative stability of emulsions was debated in other works where lipid oxidation was accelerated under high-pressure treatments [44,45]. Generally, when a fixed concentration of oil is maintained, the droplet surface would increase with the decrease of droplet size. This can result in higher exposure to free radicals and an increase in lipid oxidation [46]. Apparently, the large specific surface area of emulsions makes them also more predisposed to chemical degradation. Such results were also previously reported, for instance, in homogenized almond milk. Such samples showed higher values of hydroperoxide index when treated at high pressures than in untreated or heat-treated samples [47]. Apparently, high levels of applied pressure may speed-up oxygen uptake during cavitation or by overheating due to shear stress [48]. Other studies confirmed these conclusions [49,50,51]. However, this tendency can be changed and even inverted depending on the emulsion characteristics and the protective effect of interface against oxidation. Nakaya et al. [52] studied the effect of droplet size on oxidative stability of O/W emulsions. They found fine emulsion droplets were more stable to oxidation, as the concentration of emulsifier on smaller droplets is higher than that on the larger droplets. Therefore, the lipids in emulsion with smaller droplets become more stable against oxidation. They proposed the position of emulsifier molecules at the O/W interface can influence the movement of lipid molecules and accordingly change the oxidative stability [52]. Moreover, some authors reported that whey proteins themselves have antioxidant capacity protecting the emulsion from oxidation and binding some lipid oxidation products [46,53,54]. As the adsorbed whey protein, or the quantity of whey protein directly in contact with the oil phase, increases with the interfacial area, the smaller particles may be better protected by the proteins [55]. However, our study showed an increase in vitamin retention only up to 100 MPA. Further increase in microfluidizer pressure resulted in lower retention of vitamin A. In this case, the concentration of emulsifier was not sufficient to protect the bioactive inside the oil droplets. Overall, the pressure of a microfluidizer must be optimized to counterbalance its positive and negative effects on the resulting oxidative stability. This is in agreement with the results obtained in our experiment.

### 3.3. Long-Term Chemical Stability of Vitamin A Encapsulated in O/W Emulsions

The second step of this work was to investigate the effect of storage on the stability of vitamin A emulsions. Table 1 reports all the recovery values of vitamin A for a period of five weeks.

A three ways analysis of variance was conducted on the influence of three independent variables (microfluidizer pressure, the concentration of α-tocopherol, and time of storage) on the vitamin A loss during storage at 40 °C. The microfluidizer pressure and α-tocopherol content were the same as discussed before. Here we included, as the third factor of variability, the time of storage. The stability of emulsions was studied for five weeks (at 0, 1, 2, 3, 4, and 5 weeks) at 40 °C and analyzed on independent samples (for a total of 160 samples). The results of ANOVA showed that all the main effects were statistically significant at the 0.05 significant level. As shown before, the concentration of tocopherol was significant, explaining 49% of the total variance, followed by storage time (26.1%) and pressure (21.2%). More important, the analysis highlighted the significance of all the second-order interactions (between the effects of α-tocopherol and pressure, F(9,96) = 11, *p* < 0.001; between the effects of α-tocopherol and time, F(15,96) = 6, *p* < 0.001; and between the effects of pressure and time, F(15,96) = 28, *p* < 0.001; instead, the triple order interaction was not significant, F(45,96) = 1, *p* = 0.08.

**Figure 6:**
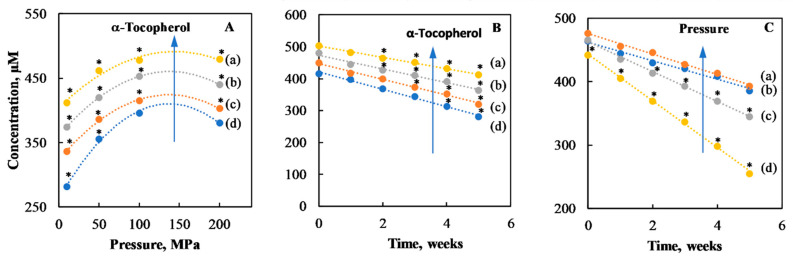
Average concentration of vitamin A showing the interaction of factors on its retention during shelf-life storage: (**A**), pressure (from 10 to 200 MPa) and α-tocopherol (from 0 to 1 mg/g); (**B**), α-tocopherol (from 0 to 1 mg/g) and time (from 0 to 5 weeks); (**C**), pressure (from 10 to 200 MPa) and time (from 0 to 5 weeks): (**a**); 0 mg/g; (**b**) 0.25 mg/g; (**c**) 0.50 mg/g; (**d**) 1.00 mg/g. Points marked with (*) are significantly different (Tukey HSD, *p* < 0.05).

Figure 6 displays the interaction effects between the three main factors taken two by two.

In detail, Figure 6A shows the interaction between applied pressures and tocopherol content. At lower α-tocopherol content, changes in pressure exert a stronger relative effect on vitamin A stability. However, at any α-tocopherol content, the maximum stability was achieved for pressures below 200 MPa. This is in agreement with the study of Hebishy et al., in which the heating of β-Lg above denaturation temperature (70 °C) can rearrange the protein molecules at the surface of the emulsion droplet. The rearrangement can result in changes of both intra- and intermolecular bonds in protein, which makes the protein interface more cohesive and prevents them from being desorbed by the surfactant [16]. Overall, the results of long-term stability confirm the preliminary conclusions achieved just after microfludization process, although, the long storage effect emphasizes the positive interaction between high pressures and high content of α-tocopherol.

Figure 6B, shows the interaction effect between α-tocopherol content and storage time. The rates of vitamin loss were 27 (d), 25 (c), 21 (b), and 17 (a) µM week-1 respectively for concentrations of α-tocopherol equal to 0.00, 0.25, 0.50, and 1.00 mg/g. Regardless of the presence of α-tocopherol, the vitamin loss showed a linear trend (*R*^2^ > 0.99) during the five weeks of storage. However, when α-tocopherol content increased, the rate of vitamin loss decreased. Finally, Figure 6C shows the interaction effect between pressure and time. When high-pressure levels were used (100 and 200 MPa), then the rate of vitamin loss was the lowest.

The optimized conditions that assure the longest storage stability of vitamin A in emulsions were calculated using the desirability plot (results not shown). The conditions that optimize the storage stability of vitamin A are (1) the highest content of α-tocopherol and (2) an intermediate high pressure between 0.86 and 1.24 MPa. The results follow the same trend as those observed just after the microfluidization process. Within such optimal conditions, the loss of vitamin A after 5 weeks of storage at 40 °C is limited to 12%.

**Figure 7:**
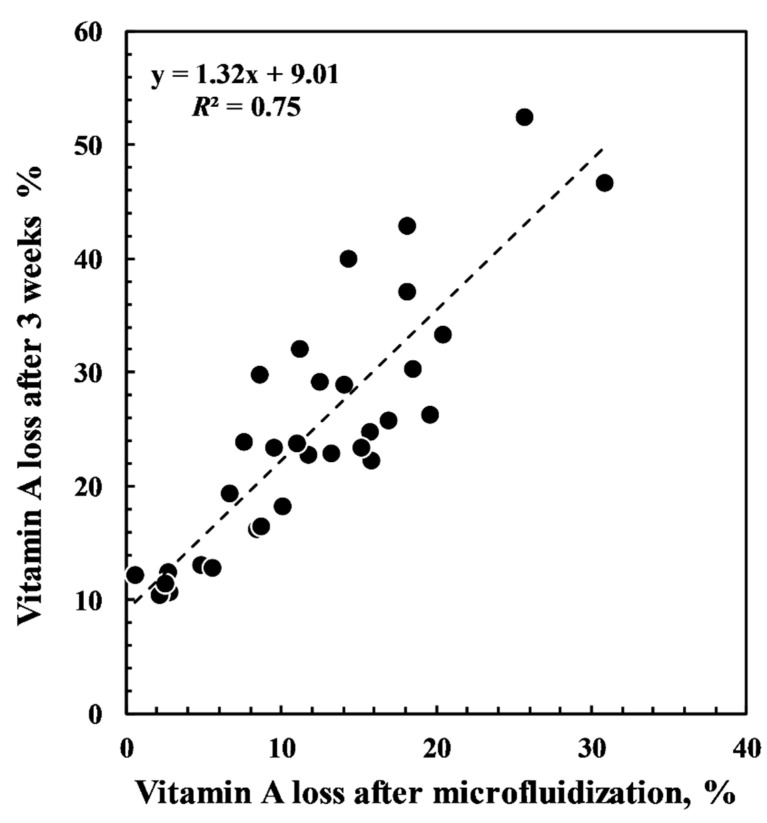
Correlation plot between percentage losses of vitamin A measured just after the microfluidizer step and after three weeks of storage.

Interestingly, the vitamin loss just after microfluidization is correlated with the loss measured during storage. After one week of storage, the correlation is *R*^2^ = 0.81, after 2 weeks it is 0.79, after three weeks it is *R*^2^ = 0.75, after four weeks it is 0.71 and, finally, after five weeks it is 0.69. Clearly, by extending the storage time, the capacity of the vitamin loss measured just after the microfluidization process of the sample is reduced. However, Figure 7 shows the correlation between vitamin A loss after microfluidization and that observed after three weeks of storage.

The results show that within three weeks of storage, the stability of the emulsion can be predicted reasonably well by simple trials performed before and after the microfluidization process. This correlation is of great importance for the food industry because it suggests the possibility to predict the long storage stability of vitamin A based on quick trials performed immediately after microfluidization.

## 4. Conclusions

In this study, vitamin A was encapsulated in O/W emulsions prepared by varying two independent factors: microfluidizer pressure and the content of α-tocopherol. The loss of vitamin A was measured after the microfluidizer and during the shelf-life study for five weeks at 40 °C. The results of ANOVA analysis showed that the α-tocopherol was the most important factor for the stability of the vitamin. In addition, the effect of microfluidizer pressure was also significant (*p* < 0.01). In the absence of α-tocopherol, the percentage loss of vitamin A was linearly decreasing by increasing the pressure from 10 to 100 MPa. However, a further increase in pressure to 200 MPa showed no improvement in the stability of vitamin A. The best-achieved conditions regarding the chemical stability of vitamin A were emulsions prepared with the maximum content of α-tocopherol (1 mg/g) and high pressure between 100–200 MPa. Within such conditions, the loss of vitamin A was limited to 12%. One further interesting finding of this study is that the stability of the emulsions in the next weeks can be to some extent predicted by testing the vitamin loss in different formulations just after the microfluidizer process. Future studies are needed to correlate the effect of pressure on the oxidative and physical stability of emulsions containing other bioactive compounds.

## Figures and Tables

**Table 1:** Three-way analysis of variance using pressure, amount of α-tocopherol, and storage time as factors. P is the microfluidizer pressure (MPa). T is the amount of α-tocopherol per g of emulsion (mg/g). The results express the concentration of vitamin A (µM) during five weeks of storage. The average precision of the results is 1%. The rate constant (k) is calculated as the best fit of a zero-order rate law and expressed as average ± standard deviation. The coefficient of determination is reported as r^2^.

		Time (Weeks)							
P	T	0	1	2	3	4	5	K	r^2^
MPa	mg/g	µM	µM	µM	µM	µM	µM	µM/day	
10	0.00	377 ± 19	357 ± 24	319 ± 26	265 ± 21	210 ± 14	167 ± 24	44 ± 12	0.982
50	0.00	424 ± 9	399 ± 1	372 ± 17	340 ± 14	315 ± 7	282 ± 10	28 ± 3	0.998
100	0.00	440 ± 4	423 ± 1	411 ± 6	393 ± 4	370 ± 14	340 ± 14	19 ± 6	0.975
200	0.00	425 ± 4	410 ± 3	374 ± 12	376 ± 15	361 ± 16	335 ± 7	17 ± 9	0.941
10	0.25	440 ± 14	392 ± 1	363 ± 13	340 ± 13	289 ± 15	226 ± 5	40 ± 14	0.974
50	0.25	455 ± 6	418 ± 2	398 ± 6	373 ± 1	347 ± 4	328 ± 15	25 ± 5	0.991
100	0.25	459 ± 6	439 ± 1	419 ± 7	405 ± 1	399 ± 1	367 ± 6	17 ± 6	0.971
200	0.25	444 ± 3	418 ± 3	417 ± 5	405 ± 4	376 ± 5	359 ± 0	16 ± 8	0.946
10	0.50	473 ± 10	422 ± 9	382 ± 3	383 ± 8	336 ± 12	290 ± 6	33 ± 14	0.960
50	0.50	480 ± 7	435 ± 14	413 ± 1	401 ± 2	381 ± 2	339 ± 11	25 ± 10	0.963
100	0.50	490 ± 14	469 ± 9	465 ± 14	448 ± 12	432 ± 5	412 ± 16	15 ± 4	0.981
200	0.50	476 ± 5	454 ± 5	446 ± 14	434 ± 6	434 ± 2	396 ± 9	13 ± 9	0.898
10	1.00	479 ± 16	451 ± 17	419 ± 13	412 ± 17	376 ± 4	336 ± 8	27 ± 9	0.976
50	1.00	504 ± 10	491 ± 11	470 ± 5	459 ± 2	435 ± 10	420 ± 3	17 ± 3	0.993
100	1.00	516 ± 8	492 ± 7	488 ± 2	465 ± 6	453 ± 11	444 ± 3	14 ± 5	0.973
200	1.00	513 ± 1	497 ± 9	483 ± 4	468 ± 4	463 ± 4	453 ± 4	12 ± 4	0.975

**Table 2:** Analysis of variance using pressure, amount of α-tocopherol, and storage time as factors. The model: Y_ijkl_ = A_jkl_ + B_ikl_ + C_ijl_ + AB_kl_ + AC_jl_ + BC_il_ + ABC_l_ + E_ijkl_.

Analysis of variance with the model: Y_ijkl_ = A_jkl_ + B_ikl_ + C_ijl_ + AB_kl_ + AC_jl_ + BC_il_ + ABC_l_ + E_ijkl_					
**Source**	**SS**	**df**	**MS**	**F**	**Sig.**
A ^1^	290269	3	96756	964	0.001
B	188085	3	62695	625	0.001
C	291593	5	58318	581	0.001
AB	13988	9	1554	15	0.001
AC	8866	15	591	5	0.001
BC	41265	15	2751	27	0.001
ABC	5987	45	133	1	0.001
Error	9629	96	100		
Total	849685	191			

1—A = α-tocopherol (µM); B = pressure of microfluidizer (MPa); C = time (weeks).

## Data Availability

The data presented in this study are available on request from the corresponding author.
